# Three-Dimensional Micro-Computed Tomography of the Adult Mouse Ovary

**DOI:** 10.3389/fcell.2020.566152

**Published:** 2020-10-19

**Authors:** Giulia Fiorentino, Annapaola Parrilli, Silvia Garagna, Maurizio Zuccotti

**Affiliations:** ^1^Laboratory of Developmental Biology, Department of Biology and Biotechnology “Lazzaro Spallanzani”, University of Pavia, Pavia, Italy; ^2^Center for Health Technologies, University of Pavia, Pavia, Italy; ^3^Center for X-ray Analytics, Empa–Swiss Federal Laboratories for Materials Science and Technology, Dübendorf, Switzerland

**Keywords:** mouse, ovary, follicles, oocyte, vasculature, micro-computed tomography, three-dimensional rendering

## Abstract

In the mouse ovary, folliculogenesis proceeds through eight main growth stages, from small primordial type 1 (T1) to fully grown antral T8 follicles. Most of our understanding of this process was obtained with approaches that disrupted the ovary three-dimensional (3D) integrity. Micro-Computed Tomography (microCT) allows the maintenance of the organ structure and a true *in-silico* 3D reconstruction, with cubic voxels and isotropic resolution, giving a precise spatial mapping of its functional units. Here, we developed a robust method that, by combining an optimized contrast procedure with microCT imaging of the tiny adult mouse ovary, allowed 3D mapping and counting of follicles, from pre-antral secondary T4 (53.2 ± 12.7 μm in diameter) to antral T8 (321.0 ± 21.3 μm) and *corpora lutea*, together with the major vasculature branches. Primordial and primary follicles (T1–T3) could not be observed. Our procedure highlighted, with unprecedent details, the main functional compartments of the growing follicle: granulosa, antrum, cumulus cells, zona pellucida, and oocyte with its nucleus. The results describe a homogeneous distribution of all follicle types between the ovary dorsal and ventral regions. Also, they show that each of the eight sectors, virtually segmented along the dorsal-ventral axis, houses an equal number of each follicle type. Altogether, these data suggest that follicle recruitment is homogeneously distributed all-over the ovarian surface. This topographic reconstruction builds sound bases for modeling follicles position and, prospectively, could contribute to our understanding of folliculogenesis dynamics, not only under normal conditions, but, importantly, during aging, in the presence of pathologies or after hormones or drugs administration.

## Introduction

In the adult ovary, folliculogenesis is a hormonally regulated process of growth and maturation from small primordial to fully grown antral follicles. The gonadotropin-independent initial recruitment of primordial follicles is followed, at puberty, by Follicle Stimulating Hormone (FSH) cyclic recruitment of early antral follicles (Hsueh et al., 2015), the latter enveloped within a calyx of capillaries arising from a branch of the ovarian artery (Fraser, 2006). Of these follicles, only a few, in polytocic species like the mouse, or one, in monotocic species like humans, complete their growth and are eventually ovulated. Follicle growth and correct acquisition of developmental competence occur thanks to a continuous, bi-directional, exchange of information both between the oocyte and its surrounding cumulus cells and among separate follicles (Zuccotti et al., 2011; Russell et al., 2016).

Most of our understanding of this process has been obtained with approaches that lead to the loss of the three-dimensional (3D) integrity of the organ. The ovary is disaggregated into its follicular units, which are either directly analyzed with molecular biology techniques, further matured *in vitro*, or cultured in media containing matrices with the aim of maintaining the follicle 3D organization (Belli et al., 2012; Telfer, 2019). Alternatively, the female gonad is fixed and sectioned for histological or immunohistochemical 2D studies.

A 3D reconstruction of the mammalian ovary would not only further improve our understanding of its tissue architecture, but, when combined with specific functional markers, it would help to reveal the multi-layered flow of molecular information that contributes to its biological function.

Up to date, tissue clearing combined with optical imaging allowed the acquisition of molecular details on the inside 3D organization of the ovary (Cordeiro et al., 2015; Faire et al., 2015; Feng et al., 2017; Kagami et al., 2018; Oren et al., 2018; McKey et al., 2020). However, a major drawback of this procedure remains the production of a distorted 3D model of the organ, stretched out along the *z*-axis, due to the lack of an equal resolution on the three axes (Schermelleh et al., 2010; Cremer et al., 2017).

On the contrary, tomographic methods produce a true 3D reconstruction of an object, with cubic voxels (i.e., image elements) and isotropic resolution, in other words, the space between two tomographic sections (*z*-axis) equals the resolution of the xy plane (Flannery et al., 1987; Mizutani and Suzuki, 2012). In order to reconstruct the folliculogenetic process inside the volume of the ovary, different attempts have been made using ultrasonography (Mircea et al., 2009; Ginther et al., 2014; Migone et al., 2016), micro-Magnetic Resonance Imaging (μ-MRI) (Hensley et al., 2007), laboratory-based X-ray micro-Computed Tomography (microCT) (Paulini et al., 2017), synchrotron radiation Computed Tomography (SR-CT) (Kim et al., 2012), and phase contrast Computed Tomography (PC-CT) (Pascolo et al., 2019). However, none of them, with the exception of SR-CT–a technique difficult to access–were capable to identify ovarian follicles smaller in size than those present in the pre-ovulatory compartment on the organ surface (e.g., 450 μm in diameter in rat; 10 mm in bovine). The main limit of ultrasonography, μ-MRI and PC-CT is an insufficient spatial resolution of these instruments, which is in the order of 10–100 μm/pixel; instead, whilst microCT and SR-CT can reach higher resolutions in the range of 0.35–10 μm/pixel, they mainly work when there is a marked contrast generated by differences in tissue radiopacity. For these reasons, it is not surprising that microCT has been extensively used with hard tissues, such as bones (Mussmann et al., 2017; Song et al., 2018) and teeth (Chałas et al., 2017; Batool et al., 2018; Velozo and Albuquerque, 2019), whose histological composition makes them naturally radiopaque to X-rays. On the other hand, its application on soft tissues is often restricted by the need to employ contrast agents that are largely unspecific with a partial capacity to highlight the presence of different structures within organs (Metscher, 2009; Pauwels et al., 2013; de S e Silva et al., 2015; de Bournonville et al., 2019).

In this study, we describe a method that, by combining an optimized contrast procedure with microCT imaging, allowed, for the first time, the 3D mapping and counting of follicles, from pre-antral secondary type 4 (T4) to fully grown antral type 8 (T8), together with the identification of the major vasculature branches inside the adult mouse ovary, one of the smallest among Mammals.

## Materials and Methods

### Animals

CD1 female mice were purchased from Charles River (Como, Italy). Animals were maintained under controlled conditions of 21°C, 60% air humidity and a light/dark cycle of 12:12 h. Research on mice was conducted with permission from the Ministry of Health (No. 1100/2016-PR) in accordance with the guiding principles of European (No. 2010/63/UE) and Italian (No. 26/2014) laws protecting animals used for scientific research.

### Ovaries Fixation

Ovaries of 8-week-old females were isolated and, after removal of the fat residuals, individually fixed in either Carnoy’s solution (60% Ethanol, 30% Chloroform, and 10% Glacial acetic acid) for 8 h at room temperature, or Bouin’s solution (15 mL Picric acid, 5 mL 40% Formaldehyde, 1 mL Glacial acetic acid) for 2 h at room temperature, or 4% Paraformaldehyde (PFA; 4 gr Paraformaldehyde in 100 mL 1× PBS) overnight at 4°C. The excess of fixative was washed out with its specific solvent.

### Contrast Agent Treatment

Following fixation, ovaries were individually treated at room temperature with one of the following contrast agent: Iodine tincture [70% Ethanol and 1% Iodine Tincture (10% in distilled water)], 25% Lugol’s solution (2.5 gr Potassium iodide and 1.25 gr Iodine in 100 mL distilled water), Phosphotungstic acid (0.2 gr PTA in 100 mL distilled water), or Uranyl acetate (1 gr in 100 mL distilled water). Different treatment times were tested, ranging from a minimum of 15 min up to a maximum of 8 h. Each contrast agent was washed out with its respective solvent, for a period ranging from 30 s to 15 h.

### Micro-CT Imaging

Following contrast treatment, ovaries were placed in an open nest created with orthodontic wax at the bottom of a 0.5 mL Eppendorf tubes filled with distilled water to avoid organ dehydration and shrinkage. Then, samples were scanned by microtomographic system Skyscan 1172 (Bruker MicroCT, Kontich, Belgium). To determine the best preparation protocol, comparative studies of the different preparations were performed, at a source voltage of 60 kV, 165 μA current and using a 0.5 mm aluminum filter, adopting a scanning resolution of 5 μm/pixel for nearly 25 min. Samples were rotated to 180° with a rotation step of 0.4°. Terminated the cone beam acquisition, the dataset acquired was of approximately 500 images in 16-bit tiff format. Three ovaries from three different females were fixed in 4% PFA, treated with Lugol’s solution for 3 h, washed for 15 h and scanned at a source voltage of 59 kV, 167 μA current, with a resolution of 1.5 μm/pixel for nearly 5 h. Samples were rotated to 180° with a rotation step of 0.1°. The scanning dataset consisted of about 2000 images in 16-bit tiff format. The final microCT sections were reconstructed using the NRecon software (Bruker MicroCT, Kontich, Belgium) in 8-bit jpg format (2000 × 2000 pixels). Sections were visualized with Fiji ImageJ (NIH) (Schindelin et al., 2012) and follicles and blood vessels were manually segmented and isolated, combing the use of regions of interest (ROIs) manager, thresholding and logical operations. 3D rendering of the follicular and vascular components was obtained with the volren module of the Avizo-9 software (Thermo Fisher Scientific) that approximates the organ isosurface from the segmented perimeters, resulting in a final model with a shaded surface and optimized transparency/opacity values.

### Histology

Histology was used as a cross-checking technique to validate the results of the microCT analysis. After microCT scanning, samples were individually dehydrated through an ascending ethanol series, clarified with xylene and embedded in paraffin wax (Paraplast Plus, Merck). Sagittal serial 6-μm cross sections of the whole ovary were prepared using a RM2125RT microtome (Leica Biosystems) and, then, were stained with Hematoxylin and Eosin. Sections were examined and digitalized at 63× magnification, using a Leica DMi6 light microscope equipped with a motorized XY scanning stage controlled by the LAS X Navigator stitching software (Leica Biosystems).

### Follicle Classification, Counting and 3D Localization

Based on a combination of morphological parameters and the size in diameter, follicular structures observed through the microCT sections of each ovary were individually counted and assigned to a specific follicle type (T4, T5, T6, T7, or T8) or *corpus luteum*. The morphological features considered were the presence/absence and size of the antrum and the thickness of the follicle cells layer surrounding the oocyte (Pedersen and Peters, 1968), but also the presence/absence of a well-visible zona pellucida space. Using Fiji ImageJ software on microCT sections, the follicle size was recorded at the major diameter and compared to that measured on histological sections prepared after microCT acquisition. More specifically, follicles were classified as T8 when they showed a size in diameter within the range of 321.0 ± 21.3 μm, combined with the presence of a large single antrum cavity, a cumulus stalk, and a well-visible zona pellucida space in between the oocyte and the surrounding cumulus cells; T7 displayed a 218.9 ± 36.9 μm diameter, a single antrum cavity smaller than T8, the presence of a cumulus oophorus and a zona pellucida space; T6 exhibited a 137.0 ± 24.9 μm diameter, several scattered small antral cavities and a zona pellucida space; T5 showed a 89.0 ± 11.2 μm diameter, the absence of antrum cavities, and the presence of a zona pellucida space separating the oocyte from the surrounding granulosa cell layers; T4, the smallest identifiable follicles by microCT, had a size in diameter of 53.2 ± 12.7 μm, they lacked of a zona pellucida space and showed a lower radiopacity compared to that of the other follicle types.

The spatial reference system used for follicle localization within each ovary was the anatomical orientation of the organ in relation to the anterior–posterior and dorsal-ventral body axes. The series of microCT sections of each ovary was divided into two halves along the dorsal-ventral axis, obtaining a dorsal and a ventral subset. Using Fiji ImageJ, each subset was partitioned into four, equally distributed, sectors, of which I and IV were positioned on the anterior region, while II and III on the posterior. A follicle was attributed to a specific sector when >50% of its volume belonged to that sector.

### Statistical Analysis

Differences in total follicles number or in the distribution of each follicle type along the anterior–posterior or dorsal-ventral axes were evaluated using the Student’s *t*-test. Differences in total follicles number or in the number of each follicle type present in the eight dorsal and ventral sectors were estimated using the one-way ANOVA followed by the Bonferroni’s *post-hoc* test. All statistical analyses were performed using the RStudio software (RStudio, Inc., version 1.1.423). Data were considered significantly different when *p* < 0.05.

## Results

Three different fixative types [Carnoy, Bouin’s solution or 4% Paraformaldehyde (PFA)] and four contrast agents (Iodine tincture, Phosphotungstic acid, Uranyl acetate or Lugol’s solution) were tested in preparation to microCT analysis. Of the three fixatives, independently of the contrast agent used, Carnoy caused a shrinkage of the ovary, which persisted even during the microCT scanning period and led to the acquisition of fuzzy images (data not shown). The Bouin’s solution, although it well preserved the histological organization, reduced tissue accessibility, resulting in an uneven contrast agent penetration and patchy images (data not shown). On the contrary, 4% PFA, a water-based fixative, turned out to be the best compromise as it maintained a good histology and it enabled an optimal contrast agent diffusion.

Following PFA fixation, treatment with the contrast agent was performed for different incubation periods, ranging from 15 min to 8 h, followed by a washing period ranging from 30 s to 15 h. Compared to either Iodine tincture (Supplementary Figure S1A), Phosphotungstic acid (Supplementary Figure S1B) or Uranyl acetate (Supplementary Figure S1C), treatment with Lugol’s solution for 3 h, followed by a washing period of 15 h, produced the best results (Supplementary Figure S2A). Contrast was homogeneous throughout the whole organ volume, highlighting, on microCT images acquired at 5 μm/pixel resolution, distinctly marked follicles. Supplementary Figure S2B shows the corresponding histological cross-section, and Supplementary Figures S2C,D are enlargements of microCT and histological images, respectively.

**FIGURE 1:**
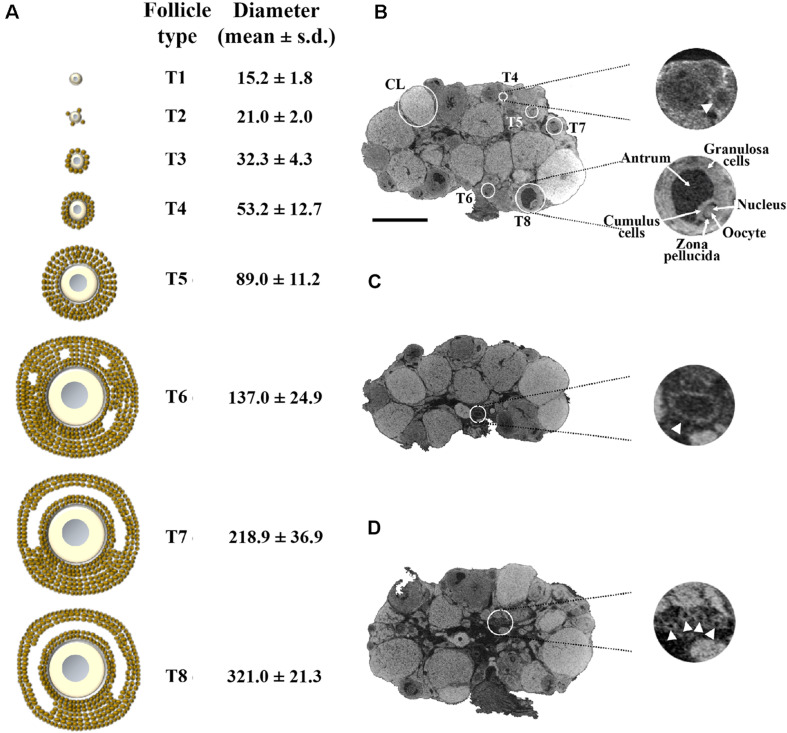
Representative 1.5 μm/pixel microCT image of the mouse ovary. **(A)** Schematic representation of the eight main stages of the mouse folliculogenesis, with their corresponding size in diameter (mean ± s.d.), during the pre-antral (T1–T5) and antral (T6–T8) growth phases. **(B)** A representative microCT section highlighting follicles from T4 to T8 and *corpora lutea* (CL). On the right-hand side (top), an enlargement of a secondary T4 follicle (arrow); on the right-hand side (bottom), an enlargement of a fully grown T8 follicle, in which the different cytological components (granulosa cells, antrum, cumulus cells, zona pellucida space, oocyte with its nucleus) are clearly visible. **(C)** microCT section with an enlargement of a vessel (arrowhead; 150 μm in diameter) at the ovarian hilum site. **(D)** microCT section with an enlargement of a group of small vessels (arrowheads; 35 μm in diameter) in the medulla region. Bar, 500 μm.

Using this latter protocol, we prepared a new group of three ovaries, isolated from three adult females, and performed microCT scanning at 1.5 μm/pixel resolution, the highest obtainable with the instrument used (Supplementary Movie S1). This resolution allowed the identification of all the phases of follicle growth. Figure 1A shows the eight main stages of mouse folliculogenesis (Pedersen and Peters, 1968; Peters, 1969), from the resting pool of tiny primordial type 1 (T1) and type 2 (T2), followed by the primary type 3 (T3), the secondary T4 through to fully grown antral T8 follicles.

Figure 1B shows a representative microCT image of an ovary in which we could pinpoint equatorially sectioned follicles ranging from the small secondary T4, with 60 μm size in diameter, through all the growing stages until the largest T8 and the *corpora lutea* (CL). In T8 follicles, the Lugol’s radiopacity brought up differences in density among the follicle’s internal components (Figure 1B, enlargement), thus allowing microCT imaging of the layers of granulosa cells, antrum, cumulus cells, zona pellucida, and of the oocyte with its nucleus. Nuclei regions were clearly seen when watched through a sequence of microCT images (Supplementary Movie S2).

A further observation that the proposed protocol allowed was the identification of the main ovarian vasculature branches, ranging from the largest vessels at the ovarian hilum site (150 μm in diameter; Figure 1C) to smaller present in the medulla region (35 μm in diameter; Figure 1D).

**FIGURE 2:**
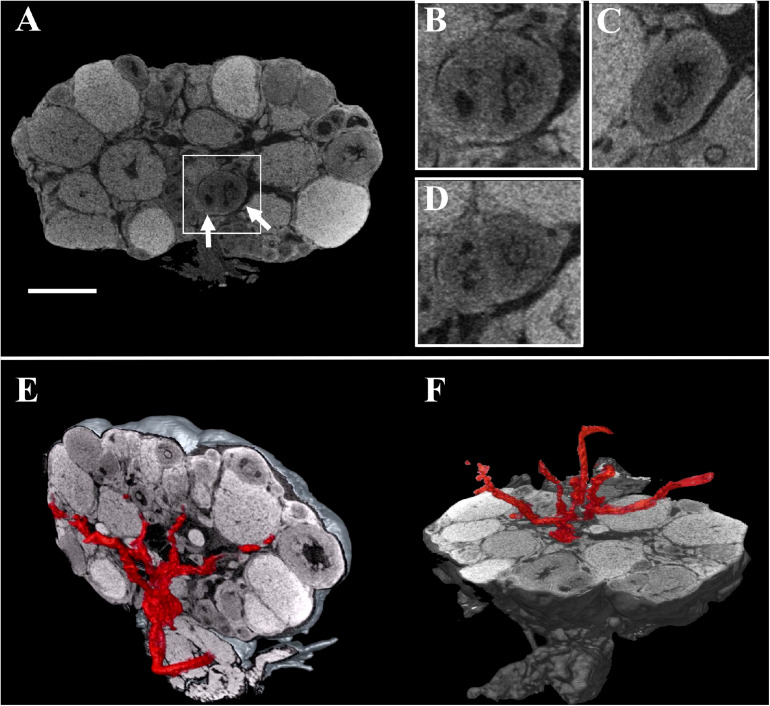
Combined three-dimensional rendering of microCT sections of the ovary and of the main vasculature. **(A)** A representative equatorial microCT slice. Inset, two T7 follicles (arrows). **(B–D)** coronal, sagittal, and axial planes of the two T7 follicles shown in the inset. **(E,F)** Reconstruction of the main vasculature on the coronal and axial section, respectively. Bar, 500 μm.

These digital microCT images were further analyzed to visualize each single follicle along the three orthogonal sections (Figures 2A–D) and to reconstruct, with a volume rendering software, a 3D model of the ovary which combined both follicular and vascular components (Figures 2E,F).

**FIGURE 3:**
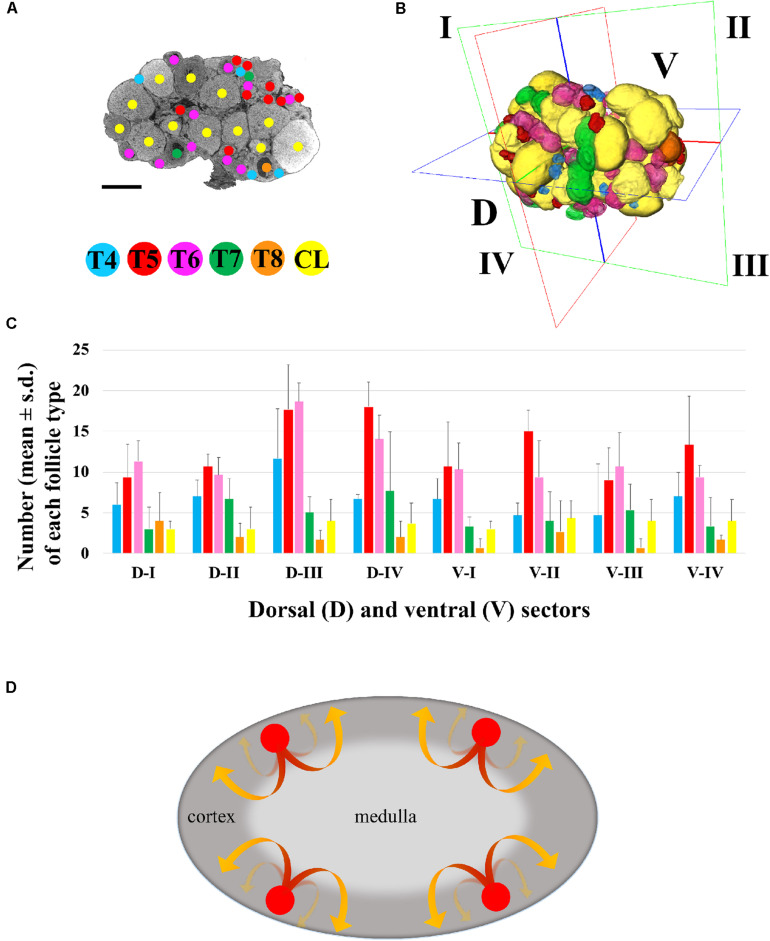
Three-dimensional mapping of growing type 4 to type 8 follicles and *corpora lutea*. **(A)** Representative microCT image of T4-CL mapping procedure, performed through all ovary sections. Bar, 500 μm. **(B)** Volume rendering of T4–T8 follicles and CL present in the ovary, and virtual segmentation of the organ’s dorsal (D-I to D-IV) and ventral (V-I to V-IV) side into four equal sectors. **(C)** Histogram describing the distribution of each follicle type and *corpora lutea* into the four dorsal and ventral sectors of three ovaries from three different female mice. **(D)** Graphical model describing the trajectory of follicles recruitment and growth. Follicle recruitment (red circle) occurs simultaneously in the cortex of all the eight sectors in which the ovary was virtually segmented. Then, follicles initiate growth toward the medulla (red segment of the arrow), increasing their size until they re-emerge into the cortical region (yellow segment of the arrow) from where, as fully grown antral follicles, they could undergo ovulation.

The possibility to rapidly watch through the entire sequence of thin 1.5 μm sagittal images offered the opportunity not only to draw, on each single section, a detailed territorial map of the follicles, from the small T4 to the largest T8 and CL (Figure 3A), but also to render their spatial 3D localization (Figure 3B) and perform a precise follicle counting.

The total number of T4-CL follicles counted in the ovary was 334.0 ± 48.8, with T5 and T6 as the most abundant (Supplementary Table S1). When the ovary was virtually divided into two equivalent halves along the anterior–posterior axis, we counted the same total number of follicles in the anterior (162 ± 34.4) and posterior (172 ± 14.4) region (*p* > 0.05), and an even distribution (*p* > 0.05) of each follicle type (data not shown). Similarly, when the ovary was halved along the dorsal-ventral axis, the number of both total and of each specific follicle type was equally allocated between the two regions (Supplementary Table S1), although dorsally we recorded a slight, but not significant (*p* > 0.05), higher total number.

Then, we proceeded by segmenting both the dorsal (D-I to D-IV) and ventral (V-I to V-IV) region into four, equally partitioned, sectors. Sectors contained an equal total number of follicles (41.7 ± 12.6; *p* > 0.05) and an identical number of each follicle type, with the exception of T5 and T6, which resulted the most numerous (*p* < 0.05), and of fully grown T8, the smallest group (Supplementary Table S1 and Figure 3C).

## Discussion

This study describes a microCT procedure which allows the observation of the inside of the tiny mouse ovary with its main ovarian artery branches, and the mapping of the 3D position of all the follicle types from T4 through T8 and *corpora lutea*, with unprecedented precision. This procedure is robust, has the advantage of being faster than other classical methods, like histology or tissue clearing (Cordeiro et al., 2015; Faire et al., 2015; Feng et al., 2017; Kagami et al., 2018; Oren et al., 2018; McKey et al., 2020), and gives a true isotropic 3D reconstruction (Flannery et al., 1987; Mizutani and Suzuki, 2012). A typical experiment requires a total of 35 man/h from ovaries isolation to completion of X-ray scanning, and 24 man/h for follicles classification and mapping.

The reason why we could not identify follicles smaller than T4 may depend on the reduced size of its enclosed oocyte (<20 μm in diameter)–much similar to that of the companion follicle cells–and on the absence around the germ cell of a zona pellucida, which begins to be fully present in T4 follicles, and that, with its glycoprotein layer, may create the right conditions for the contrast agent to be deposited. To this end, we are now working on improving the contrast procedure.

Besides the observation of growing follicles, our tomographic procedure allowed–to the best of our knowledge for the first time–the identification of the main functional cellular and subcellular compartments that constitute the growing follicle. Thus, the granulosa, antrum, cumulus cells, zona pellucida, and oocyte together with its nucleus, were clearly brought up not only in the largest T8 follicles, but also, when present, in smaller follicle types. The observation of a subcellular component such as the nucleus might be explained with an on-site specific contrast created by a differential local density of a tightly dense nucleolus surrounded by centromeric heterochromatin (Longo et al., 2003).

The *in-silico* reconstruction of the ovary with isotropic resolution allowed a 360° observation along the three axes and an accurate counting of each T4 to T8 follicles and CL comprised dorsally and ventrally. Our results highlight a homogeneous distribution of all the different follicle types not only between the dorsal and ventral region, but also among the eight sectors in which they were divided. In other words, each sector houses an equal number of follicles, including CL and, also, each follicle type is equally represented, with T5/T6 and fully grown T8 being the most and least abundant, respectively. Taken as a whole, these data suggest that (i) follicle recruitment is homogeneously distributed all-over the cortex, (ii) it occurs simultaneously in all the eight sectors, and (iii) follicles initiate and terminate their growth within the same sector (Figure 3D). After recruitment, biomechanical signals might guide follicles growth toward the less stiff environment of the medulla (Shah et al., 2018) until when they locally re-emerge, tens of folds bigger in size (T8), on the cortex surface from where they could be ovulated, leaving the *corpora lutea* behind.

In conclusion, the quantitative topographic information obtained builds sound bases for modeling follicles position inside the ovary, and, prospectively, could contribute to our understanding of the dynamics of the folliculogenetic process, not only under normal conditions, but, importantly, also during aging, in the presence of pathologies (e.g., polycystic ovary syndrome, hyperstimulation syndrome or ovarian cancer) or after hormones or drugs administration. In addition, our study on the mouse ovary, which is one of the tiniest in Mammals, sets the bases for a comparative 3D anatomy within this class, but also could be extended to a reconstruction of the evolutionary history of the female gonad. Finally, microCT preserves the integrity of the sample and facilitates its further analysis with other approaches. The use of PFA well maintains both tissue histology and protein components, thus allowing additional immunohistochemical investigations into more specific molecular functional markers.

## Data Availability Statement

The raw data supporting the conclusions of this article will be made available by the authors, without undue reservation.

## Ethics Statement

The animal study was reviewed and approved by the Animal Welfare Committee of the University of Pavia.

## Author Contributions

GF prepared all the samples used for microCT and histological analyses, and did the follicle counting. AP designed the microCT analysis and performed the scanning of the samples. GF and AP did the post-microCT software analysis and 3D rendering. SG and MZ designed, followed, and interpreted the experiments and together with GF and AP wrote the manuscript. All authors contributed to the article and approved the submitted version.

## Conflict of Interest

The authors declare that the research was conducted in the absence of any commercial or financial relationships that could be construed as a potential conflict of interest.

## Supplementary Material

Supplementary Figure 1 Representative 5 μm/pixel microCT images of PFA-fixed mouse ovaries treated with different contrast agents. **(A)** Iodine tincture. **(B)** Phosphotungstic acid. **(C)** Uranyl acetate. Bar, 500 μm.

Supplementary Figure 2 Representative 5 μm/pixel microCT image of the mouse ovary after PFA fixation and contrast with Lugol’s solution. **(A)** A representative microCT image acquired at 5 μm/pixel clearly showing various *corpora lutea* (CL), type 7 (T7), type 6 (T6), and type 4 (T4) follicles; Bar, 500 μm. **(B)** Hematoxylin and Eosin stained cross-section corresponding to the microCT image shown in panel **(A)**; Bar, 500 μm. **(C,D)** Enlarged details of panels **(A,B)**, respectively; Bar, 150 μm.

Supplementary Movie 1 A representative microCT imaging of an adult mouse ovary.

Supplementary Movie 2 MicroCT identification of the oocyte’s nucleus inside two type 7 follicles.
