# Supplementary figures and images for: Three-Dimensional Micro-Computed Tomography of the Adult Mouse Ovary
Source: Front Cell Dev Biol. 2020 Oct 19;8:566152. doi: 10.3389/fcell.2020.566152 (PMC7604317; doi:10.3389/fcell.2020.566152)

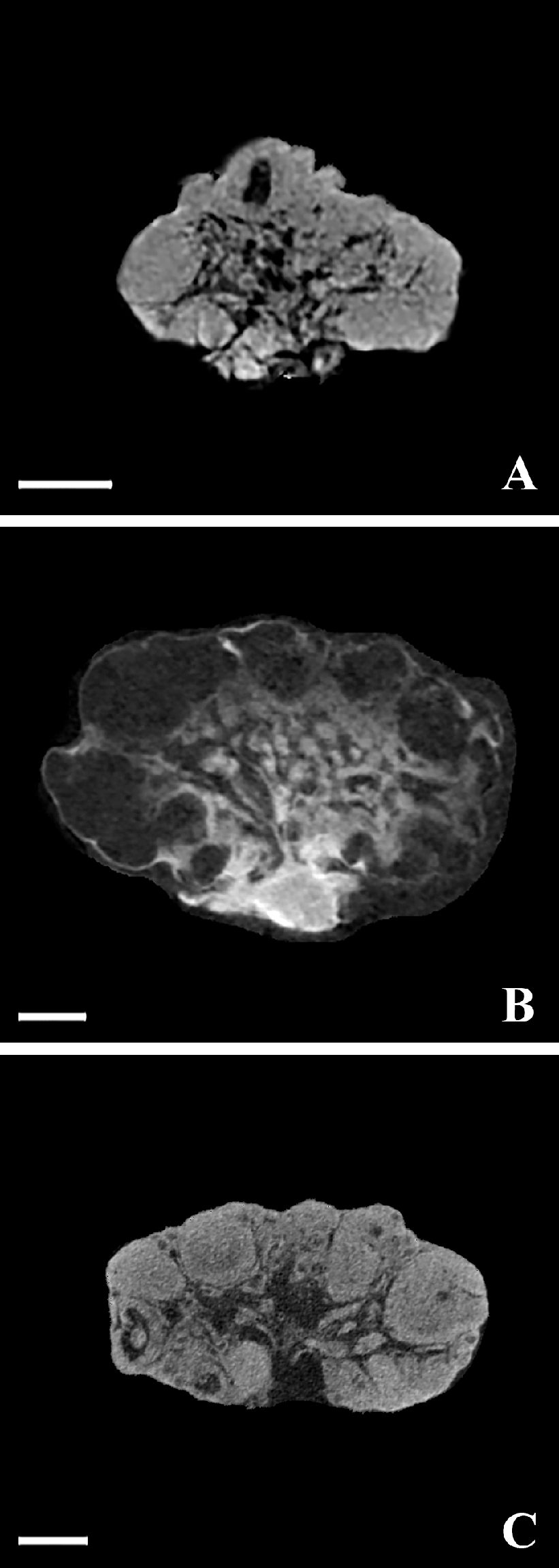

Supplement: Supplementary Figure 1 — Representative 5 μm/pixel microCT images of PFA-fixed mouse ovaries treated with different contrast agents. (A) Iodine tincture. (B) Phosphotungstic acid. (C) Uranyl acetate. Bar, 500 μm. [file Image_1.TIF]

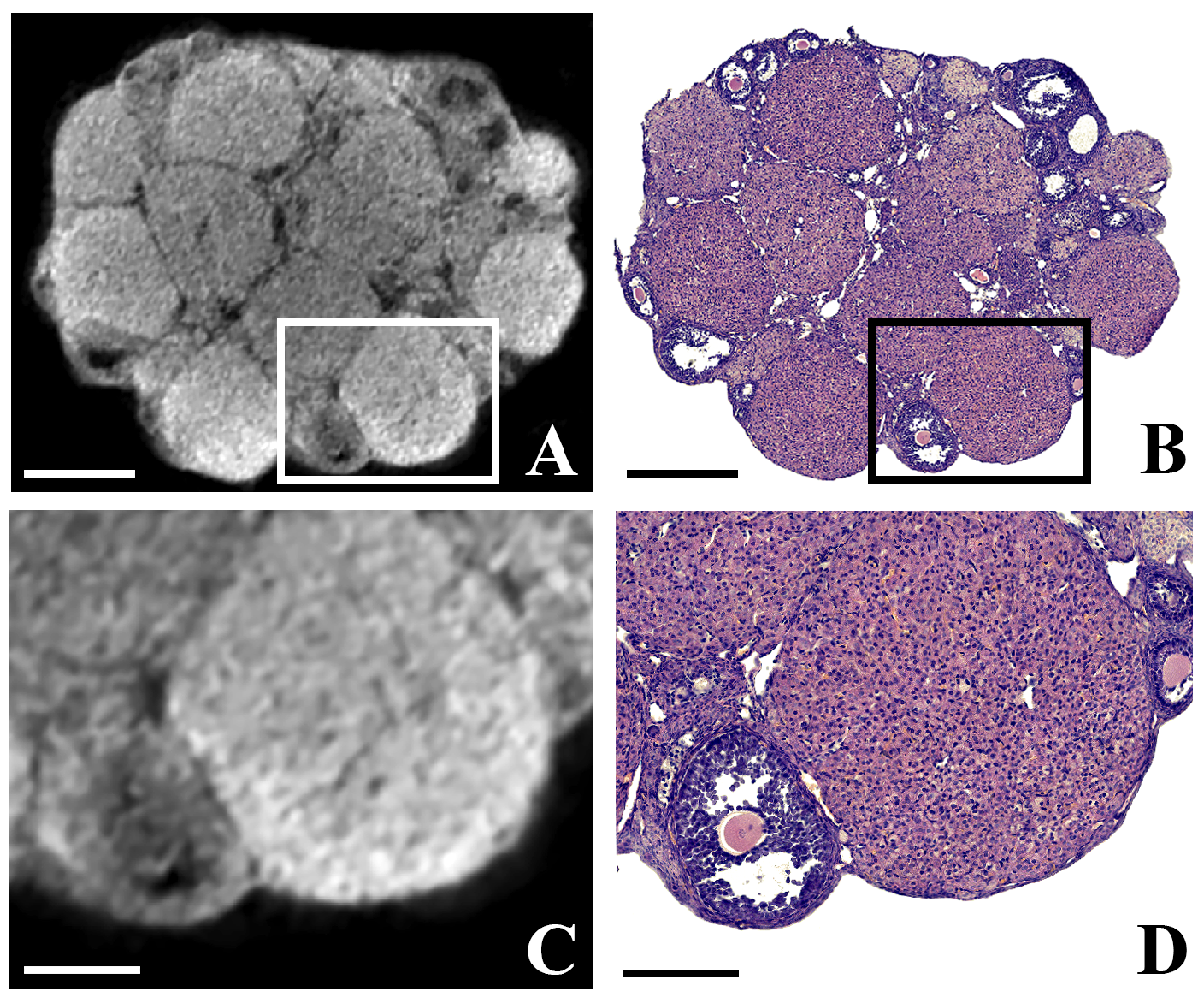

Supplement: Supplementary Figure 2 — Representative 5 μm/pixel microCT image of the mouse ovary after PFA fixation and contrast with Lugol’s solution. (A) A representative microCT image acquired at 5 μm/pixel clearly showing various corpora lutea (CL), type 7 (T7), type 6 (T6), and type 4 (T4) follicles; Bar, 500 μm. (B) Hematoxylin and Eosin stained cross-section corresponding to the microCT image shown in panel (A); Bar, 500 μm. (C,D) Enlarged details of panels (A,B), respectively; Bar, 150 μm. [file Image_2.TIF]
