# Supplementary material for: Three-Dimensional Micro-Computed Tomography of the Adult Mouse Ovary
Source: Front Cell Dev Biol. 2020 Oct 19;8:566152. doi: 10.3389/fcell.2020.566152 (PMC7604317; doi:10.3389/fcell.2020.566152)
Supplement: Supplementary file 5 [file Table_1.docx]

**Table S1.** Follicle type number (mean+s.d.) counted in each of the four dorsal (D-I to D-IV) and ventral (V-I to V-IV) sectors in which the three ovaries studied were sectioned. Statistical analysis with Student’s t-test of the number of follicles present in the dorsal or ventral regions did not show significant differences (*p* > 0.05). Statistical analysis with one-way ANOVA followed by the Bonferroni’s post-hoc test performed comparing the number of each follicle type among the eight sectors (horizontal comparison) did not highlight significant differences (*p* > 0.05). Instead, the same analysis, when done comparing follicle types within the same ovarian sector (vertical comparison), brought up the following significant differences (*p* < 0.05): D-I, no significant differences; D-II, T5 vs T8, T5 vs CL, T6 vs T8, T6 vs CL; D-III, T5 vs T8, T5 vs CL, T6 vs T8, T6 vs CL; D-IV, T4 vs T5, T5 vs T8, T5 vs CL, T6 vs T8; V-I, T5 vs T8, T6 vs T8; V-II, T4 vs T5, T5 vs T7, T5 vs T8, T5 vs CL; V-III, no significant differences; V-IV, T5 vs T7, T5 vs T8. Also, the comparison of the total number of each follicle type present in the ovary showed significant differences between T5 *vs* T7 and T5 *vs* T8.

| **Follicle type** | | **Total number follicles** | **Dorsal** | | | | | **Ventral** | | | | |
| --- | --- | --- | --- | --- | --- | --- | --- | --- | --- | --- | --- | --- |
|  |  |  | 186.3+32.5 | | | | | 147.7+18.6 | | | | |
|  |  |  | **D-I** | **D-II** | **D-III** | **D-IV** | **V-I** | | **V-II** | **V-III** | **V-IV** |  |
| **Total** | 334.0+48.8 | | 36.7+12.1 | 39.0+5.3 | 58.7+4.2 | 52.0+12.3 | 34.7+10.0 | | 40.0+11.53 | 34.3+14.6 | 38.7+14.6 |  |
| **T4** | 54.3+12.2 | | 6.0+2.6 | 7.0+2.0 | 11.7+6.1 | 6.7+0.6 | 6.7+2.5 | | 4.7+1.5 | 4.7+6.3 | 7.0+3.0 |  |
| **T5** | 103.7+20.1 | | 9.3+4.0 | 10.7+1.5 | 17.7+5.5 | 18.0+3.0 | 10.7+5.5 | | 15.0+2.6 | 9.0+4.0 | 13.3+6.0 |  |
| **T6** | 93.3+14.2 | | 11.3+2.5 | 9.7+2.1 | 18.7+2.3 | 14.0+3.0 | 10.3+3.2 | | 9.3+4.5 | 10.7+4.2 | 9.3+1.5 |  |
| **T7** | 38.3+23.4 | | 3.0+2.6 | 6.7+2.5 | 5.0+2.0 | 7.7+7.2 | 3.3+1.1 | | 4.0+3.6 | 5.3+6.2 | 9.3+3.5 |  |
| **T8** | 15.3+8.5 | | 4.0+3.5 | 2.0+1.7 | 1.7+1.1 | 2.0+2.0 | 0.7+1.1 | | 2.7+3.8 | 0.7+1.1 | 1.7+0.6 |  |
| **CL** | 29.0+4.4 | | 3.0+1.0 | 3.0+2.6 | 4.0+2.6 | 3.7+2.5 | 3.0+1.0 | | 4.3+2.1 | 4.0+2.6 | 4.0+2.6 |  |
